# Design of chimeric antigen receptors with integrated controllable transient functions

**DOI:** 10.1038/srep18950

**Published:** 2016-01-11

**Authors:** Alexandre Juillerat, Alan Marechal, Jean-Marie Filhol, Julien Valton, Aymeric Duclert, Laurent Poirot, Philippe Duchateau

**Affiliations:** 1 Cellectis Inc, 430E, 29th street, NYC, NY 10016, USA; 2 Cellectis SA, 8 rue de la croix Jarry, 75013 Paris

## Abstract

The ability to control T cells engineered to permanently express chimeric antigen receptors (CARs) is a key feature to improve safety. Here, we describe the development of a new CAR architecture with an integrated switch-on system that permits to control the CAR T-cell function. This system offers the advantage of a transient CAR T-cell for safety while letting open the possibility of multiple cytotoxicity cycles using a small molecule drug.

Adoptive immunotherapy using engineered T-cells has emerged as a powerful approach to treat cancer. The potential of this approach relies on the ability to redirect the specificity of T cells through genetic engineering and transfer of chimeric antigen receptors (CARs) or engineered TCRs^1^. Numerous clinical studies have demonstrated the potential of adoptive transfer of CAR T cells for cancer therapy^2^,^3^,^4^,^5^ but also raised the risks associated with the cytokine-release syndrome (CRS) and the “on-target off-tumor” effect^3^,^6^,^7^,^8^. To date, few strategies have been developed to pharmacologically control CAR engineered T-cells and may rely on suicide mechanisms^9^,^10^,^11^,^12^,^13^,^14^. Such suicide strategies leading to a complete eradication of the engineered T-cells will result in the premature end of the treatment. Consequently, implementing non-lethal control of engineered CAR T-cells represents an important advancement to improve the CAR T-cell technology and its safety. Small molecule based approaches that rely on dimerizing partner proteins have already been used to study, inter alia, the mechanism of T-cell receptor triggering^15^. Very recently, Lim and colleagues have adapted this approach to control engineered T-cells through the use of a multichain receptor^16^.

Here, we describe a strategy to create a switchable engineered CAR T-cells. Our approach is based on engineering a system that is directly integrated in the hinge domain that separate the scFv from the cell membrane. In addition, we chose to implement this strategy in a novel CAR architecture that relies on the FceRI receptor scaffold. The particularity of this design reside in the possibility to split or combine different key functions of a CAR such as activation and costimulation within different chains of a receptor complex, mimicking the complexity of the TCR native architecture. In this report, we showed that the hinge engineering approaches allowed to turn a T-cell endowed with an engineered CAR from an off-state to an on-state. By controlling the scFv presentation at the cell surface upon addition of the small molecule, our system allowed to further induce the cytolytic properties of the engineered T-cell. Overall, this non-lethal system offers the advantage of a “transient CAR T-cell” for safety while letting open the possibility of multiple specific cytotoxicity cycles using a small molecule drug.

## Results

### Experimental setup and CAR architecture

**Figure 1:**
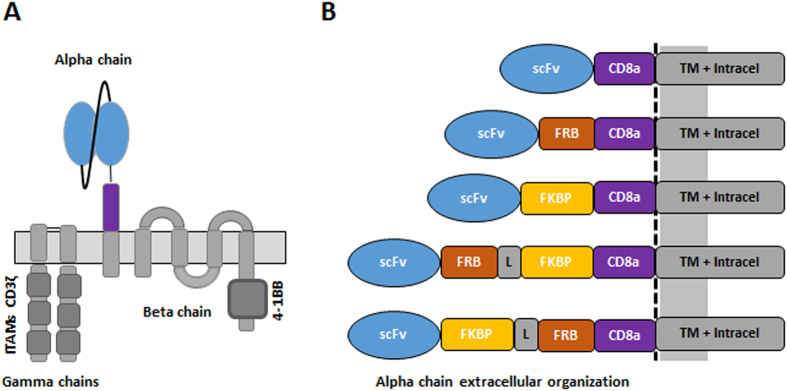
Schematic representation of the engineered mcCAR and assessment of the small molecule switch-on. (**A**) Organization of the engineered mcCAR based on FcεRI. (**B**) Design of the hinge domains that incorporate FRB and/or FKBP domains.

The CAR T-cell performance is intimately linked to an optimal interaction of the scFv to the targeted antigen. We thus conceived a system where controlled of the hinge that separates the scFv from the cell membrane could be obtained upon addition of a small molecule. As a first proof of concept, we focused on the well described and widely used macrolide rapamycin that binds with high affinity to the FKBP12 protein, creating a complex that subsequently interacts with a domain of mTOR (FKBP-rapamycin binding, FRB)^17^,^18^. In addition, we chose to implement this molecular switch strategy in a novel CAR architecture based on the FcεRI receptor scaffold, an oligomeric complex composed of three different polypeptide chains (alpha, beta and gamma)^19^,^20^. The native activation domains on the gamma and beta subunits were substituted by the intracytoplasmic signaling region of the ζ-chain of the CD3–T cell receptor and by the signaling domains from co-stimulatory 4-1BB (CD137) respectively. The extracellular domain of the alpha subunit was substituted by a single-chain variable fragment (scFv) targeting the well documented CD19 antigen fused to a hinge domain derived from the T-cell surface glycoprotein CD8 alpha chain (CD8a) (Fig. 1A)^21^,^22^. Our strategy was comforted by very recent studies that have reported such approach to create engineered mutichain receptors with novel possibilities to control and improve the efficiency of CAR T-cells^16^,^23^.

### Engineered CAR T cells are responsive to addition of a small molecule

**Figure 2:**
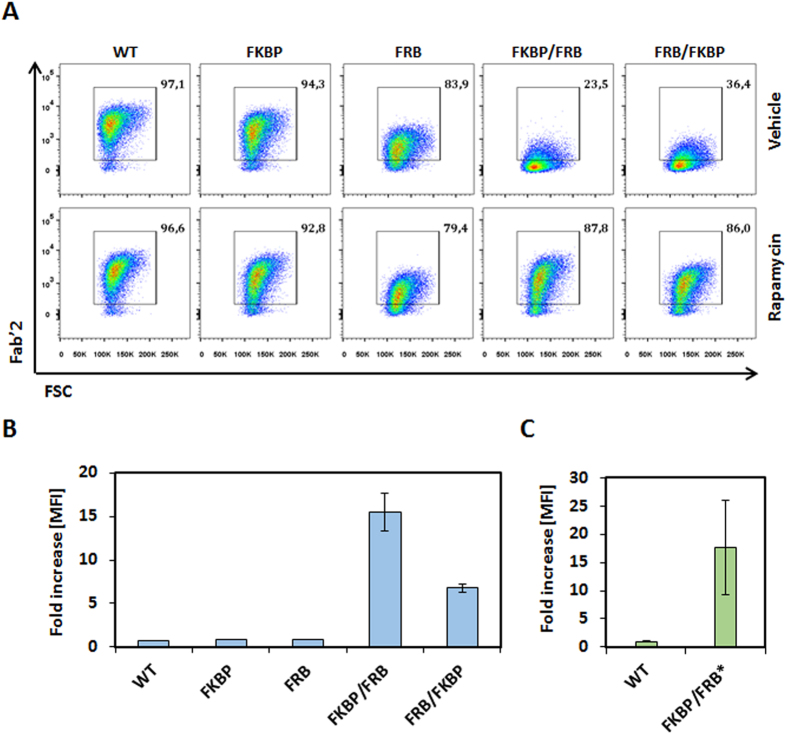
Surface detection of the engineered CAR in response to small molecules. (**A**) Percentages of live cells positive for surface detection of mcCAR in function of presence of vehicle (DMSO) or rapamycin. The detection of the Fab’2 region of the scFv is shown in a representative experiment. (**B**) Fold increase of the median fluorescence intensity (MFI) upon addition of the small molecule rapamycin as depicted in the whole live cell population. (**C**) The fold increase in the median fluorescence intensity (MFI) upon addition of the small molecule AP21967 is depicted using the T2098L mutant FKBP/FRB* construct. N = 2, error bars denote s.d.

To design an integrated system to switch the scFv/antigen interaction between on/off states, we inserted either the FRB, the FKBP12, or fusion of the FRB and FKBP12 between the CD8a hinge and the scFv domains (Fig. 1B and Supplementary Table 1). As a starting experiment, we transfected primary T cell with mRNAs encoding each chain of the multichain CAR (mcCAR). Upon addition of rapamycin, we monitored changes in the detection of the extracellular hinge domain by tracking the Fab’2 domain of the CD19-targeting scFv (100 nM, 20 h). First in absence of the small molecule (rapamycin), we found that a high level of surface detection could only be achieved for the wild type mcCAR and the FKBP-mcCAR with above 90% of positive cells with an overall high MFI (Fig. 2A). For the FRB-mcCAR, the percentage of positive cells was also high (above 80%) but with a lower MFI (3–5 fold decreased when compared to the mcCAR or FKBP-CAR), which might have resulted from a destabilization of the chain due to the FRB being out of its native context (mTOR). The presence of both FKBP and FRB in the stalk region virtually abolished surface detection of the scFv, independently of their reciprocal position (below 40% of positive cells, with up to 40 fold decrease in MFI when compared to the mcCAR). Interestingly, while the addition of rapamycin barely affected the mcCAR, FRB-mcCAR and FKB-mcCAR constructs, when considering the percentage of positive cells or the MFI (Fig. 2A,B and Supplementary Fig. 1), it strongly improved (up to 15 fold when considering the MFI and 3 fold when considering the percentage of positive cell) the surface detection of the FKBP/FRB-mcCAR and FRB/FKBP-mcCAR constructs (Fig. 2A,B), turning the system from an off to an on state. This variation of detection upon addition of rapamycin may results from different factors, including stabilization of the CAR chain that is containing the switch on component. However it has to be noted that the small molecule was always required to efficiently turn-on the detection of the FKBP/FRB-CAR of the CAR at the surface of the T-cell.

As an alternative to the use rapamycin-resistant T-cells (rapamycin has immunosuppressive properties)^24^, we next tested the synthetic non-immunosupressing AP21967 rapamycin synthetic analog, which binds to the FKBP12 but does not promote the binding to the FRB domain of mTOR. Accordingly, we also include the T2098L mutation in the FRB domain (referred as FRB*) to allow the FKBP/AP21967/FRB* complex to be formed^25^. We focused only on the FKBP/FRB-CAR scaffold as it showed the strongest rapamycin-induced increase in detection (Fig. 2B). Consistent with our previous results, the new construct was only strongly detected on the T-cell surface upon addition of the AP21967 drug (Fig. 2C and Supplementary Fig. 2).

**Figure 3:**
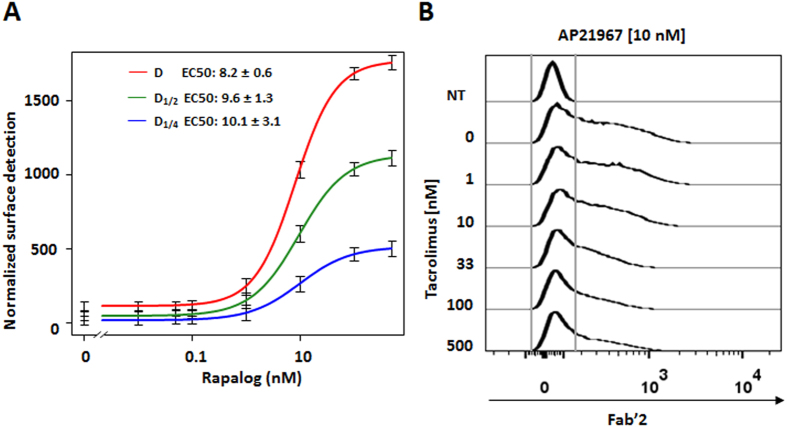
Characterization of the small molecule switch-on system. (**A**) Determination of the AP21967 EC50 with CD19 targeting engineered CAR. T-cells transfected with three doses (D, D_1/2_ and D_1/4_) of mRNA coding for the engineered CAR were treated with increasing amount of AP21967 rapamycin synthetic analog. The Fab’2 region of the scFv is detected. (**B**) Competition experiment between AP21967 (10 nM) and tacrolimus (0 to 500 nM). N = 2, error bars denote s.d.

To evaluate the AP21967 usable dose range for the switch-on system we performed a dose response assay (Fig. 3A). The results we obtained indicated a maximum signal induction at 100 nM and an EC50 value of approximately 10 nM (8.2–10.1 nM, Fig. 3A) that was independent from the amount of transfected engineered CAR. To validate the portability of the switch-on approach, we engineered a CAR targeting CD123. As demonstrated by a similar EC50 value of 10 nM (7.3–8.7 nM, Supplementary Fig. 3), we found that the nature of the scFv did not influence the switch-on properties. Remarkably, the EC50s are in range with rapamycin concentrations reported in peripheral blood or tumor tissue of patients^26^, suggesting that the switch-on system may be sensitive to clinically relevant concentration.

### Modulation of the response to AP21967 with tacrolimus

The possibility to further modulate the system using alternative small molecule competitors offers additional control of the engineered CAR T-cells. To illustrate the possibility to tune the amount of CAR locked in an on-state at the cell surface, we used tacrolimus (FK506), a small molecule known to bind to the FKBP12 without enabling to form a complex with the FRB. AP21967 (or rapamycin) and tacrolimus have identical FKBP12 binding core and should compete for the same binding site within the FKBP moiety^27^,^28^. T-cells transfected with the engineered CAR were incubated with a fixed amount of AP21967 (10 nM) and increasing amount of tacrolimus (0 to 500 nM) and the surface labelling of the scFv was recorded. As expected, addition of increasing amounts of tacrolimus competed with AP21967 for the binding site on FKBP and decreased the surface detection of the CAR (Fig. 3B).

### Engineered CAR T cells display antitumor cytotoxicity upon addition of AP21967

**Figure 4:**
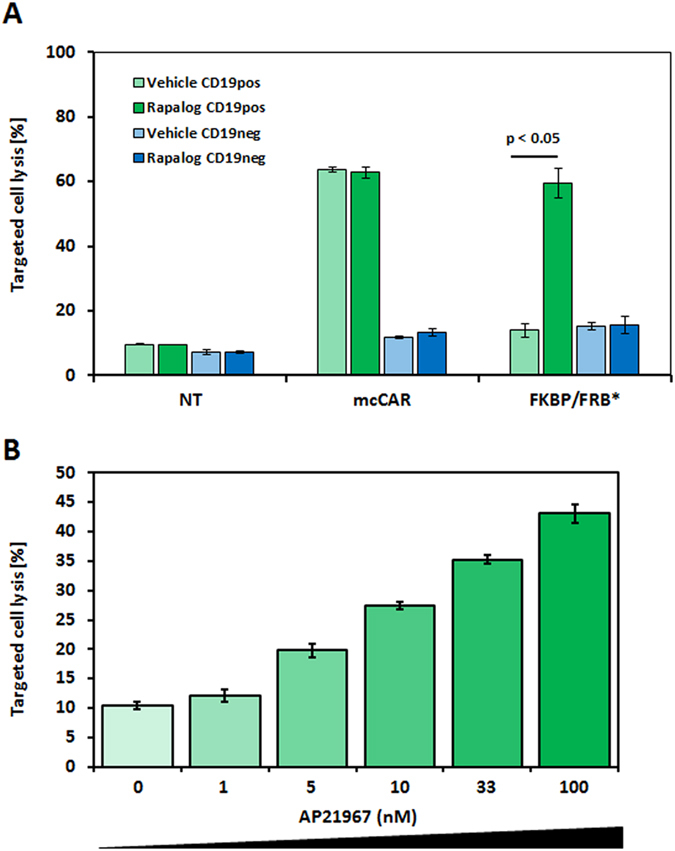
Specific cytolytic properties of the engineered CAR T-cells. (**A**) The effect of the AP21967 rapamycin synthetic analog on the cytolytic capacites of the of the CAR T cells toward model antigen presenting cell was assessed in a flow-based cytotoxicity assay. The CD19^pos^ and a CD19^neg^ target cell viability was measured after coculture with engineered CAR T-cells in presence or absence of AP21967. Effector/target ratios was set to 20:1. NT represents non-transfected T-cells, N = 2, error bars denote s.d. (**B**) The effect of the AP21967 rapamycin synthetic analog dose on the cytolytic capacites of the of the CAR T cells was measured in a flow-based cytotoxicity assay using the CD19^pos^ cell line. The Effector/target ratio was set to 10:1. The experiment was done in triplicate, error bars denote s.d.

We next intended to illustrate that the cytotoxicity of a T-cell expressing such engineered CAR can be controlled in line with its surface presentation. Therefore, we evaluated *in vitro* the cytolytic capacities of CAR T-cells toward a CD19^pos^ and a CD19^neg^ model cell line, using flow-based assay^29^. Remarkably, the results we obtained showed that the engineered FKBP/FRB-CAR T-cell presented a significant cell lysis activity only in presence of the AP21967 (Fig. 4A and Supplementary Fig. 4). We further showed that the hinge engineering did not impair the specificity feature of the engineered T-cell as no cytotoxicity was observed on CD19^neg^ target cells (Fig. 4A). Next we performed a dose response (0, 1, 5, 10, 33, 100 nM) of the AP21967 and measured the resulting cytolytic capacities of the engineered CAR T-cells. We found that the level of target cell killing correlated, as expected, with variation of the AP21967 (Fig. 4B). We futher calculated an EC50 of approximately 10 nM (12.7 nM), in range of the one determined using the surface detection. The level of targeted cell killing also correlated with the level of CAR detection (Supplementary Fig. 5).

Altogether, the results presented here provide the proof of principle of engineering the hinge domain of a CAR molecule to create an integrated switch-on system for logic gating strategies. Beyond these first steps, additional work will have to be performed to asses the full therapeutic potential of this technology, including inter alia (i) the stable integration of the CAR in the cell genome and (ii) a complete characterization of the AP21967 rapamycin synthetic analogue that has not been used in human.

## Discussion

The possibility to endow T cells with chimeric antigen receptors (CAR) has been described for more than two decades. Recent clinical implementation of adoptive cell transfer of CAR engineered T cells has proven a powerful and successful approach to cancer immunotherapy. The capability to control T cells endowed permanently with such molecules is a key feature to improve the safety of this technology. Within the conceptual framework of a CAR molecule, many alterations have been described to improve the capacity of engineered T cells to elicit antitumor functions *in vitro* and *in vivo*. Prior studies were in particular focused on optimizing the cytotoxic activity or proliferative capacities of CAR T-cells through engineering of the intracellular domain of the CARs, leading to the 2^nd^ and 3^rd^ generation of molecules^30^,^31^. However, the development of such highly active engineered T-cells have also raised concerns on the safety of this technology^32^. In particular, outcomes from clinical trial pointed out the cytokine-release syndrome (CRS) and the “on-target off-tumor” undesired targeting, both potentially leading to fatal issues. Therefore, increasing resources and effort have been allocated to better master the specificity and activation of engineered T-cell in a spatial and temporal manner using synthetic biology approaches^33^.

The transient surface expression of a CAR through RNA electroporation of T cells has already been demonstrated to be an alternative to permanently modified T-cells for improved safety^34^. However, such RNA-modified CAR T-cells head toward a permanent “off state” (RNA degradation and cell division). The all-in-one system presented in this report, with the switch incorporated in the CAR, possesses the advantages to be transient-like and to keep the cells alive. Therefore, it open the possibility to spatiotemporally induce multiple cytotoxicity cycles to control potential adverse effects. In addition, this switch-on CAR system is perfectly compatible with novel approaches that promote targeted control of drugs or engineered CAR-T cells delivery^35^,^36^. Finally, one could imagine developing similar strategies of CAR engineering to promote a small molecule off-switch that perturb optimal presentation of the antigen targeting moiety. In conclusion, while this work provides the basic framework to use alternative multi chain CARs, we believe that the easy-to-implement strategy described in this report is paving the way toward the development of novel more controlled and potentially safer engineered CAR T-cells.

## Methods

All individual chains of the CAR architecture were amplified by PCR using oligo pairs α-chain-F/α-chain-R, β-chain-F/β-chain-R and γ-chain-F/γ-chain-R prior to mRNA synthesis (Supplementary table 2). mRNA encoding the α-chain, β-chain or γ-chain were *in vitro* transcribed from the PCR product and polyadenylated using the mMessage mMachine T7 Ultra kit (Life technologies) following the manufacturer’s instructions. RNAs were purified with RNeasy columns (Qiagen), eluted in cytoporation medium T and quantified by measuring absorbance at 260 nm using a Nanodrop ND-1000 spectrophotometer. Quality of the RNA was verified on a denaturing formaldehyde/MOPS agarose gel. Sequences of FKBP and/or FRB domains are given in Supplementary table 1.

### Transfection

T lymphocytes were transfected by electrotransfer of messenger RNA using an AgilePulse MAX system (Harvard Apparatus) 3 to 6 days after activation. Following removal of activation beads, cells were pelleted, resuspended in cytoporation medium T at 28 × 106 cells/ml. 5 × 106 cells were mixed with the previously synthetized mRNA into a 0.4 cm cuvette. This particular setup allowed maintaining an identical stoichiometry (1 α/1 β/2 γ) of each chain for all constructs. Dose D corresponded to a total amount of mRNA of 33.5 μg (16 μg alpha chain, 7.5 μg beta chain, 10 μg gamma chain).

The electroporation consisted of two 0.1 ms pulses at 1200 V followed by four 0.2 ms pulses at 130 V. Following electroporation, cells were diluted into 2 mL culture medium and incubated at 37 °C/5% CO_2_. Vehicle (DMSO or ethanol, Sigma-Aldrich or Carlo Erba), Rapamycin (Sigma-Aldrich) or AP21697 (Clontech) was added 2 hours after mRNA electrotransfer and T-cell were incubated for 20 hours at 37 °C, CO_2_ 5%.

For competition experiments, AP21697 (10 nM) was added simultaneously with various amounts (0, 1, 10, 33, 100 or 500 nM) of tacrolimus (Sigma-Aldrich) and T-cell were incubated for 20 hours at 37 °C/5% CO_2_.

### Flow cytometry

Primary labelling for the detection of the CD19 targeting α-chain was performed with anti-Fab’2-Biotin (goat anti-mouse IgG, Fab’2 fragment specific, Jackson Immunoresearch) in PBS FBS 2%, EDTA 2 mM, azide 0.1% for 20 min at 4 °C followed by a two washing steps with PBS FBS 2% EDTA 2 mM azide 0.1%. Secondary labelling was performed with Streptavidin-APC (BD Pharmingen) in PBS FBS2% EDTA 2 mM azide 0.1% for 20 min at 4 °C followed by a washing step in PBS FBS2% EDTA 2 mM azide 0.1% and a washing step in PBS.

Primary labelling for the detection of the CD123 targeting α-chain was performed with Fc-tagged recombinant CD123 (Lake Pharma) in PBS FBS2%, EDTA 2 mM, azide 0.1% for 20 min at 4 °C followed by a two washing steps with PBS FBS2% EDTA 2 mM azide 0.1%. Secondary labelling was performed with PE labeled Goat Anti-Mouse IgG (subclasses 1 + 2a + 2b + 3), Fcγ Fragment Specific (Jackson Immunoresearch) in PBS FBS2% EDTA 2 mM azide 0.1% for 20 min at 4 °C followed by a washing step in PBS FBS2% EDTA 2 mM azide 0.1% and a washing step in PBS. Following the extracellular labelling, the cell viability was monitored using the efluor450 or efluor780 (ebioscience) in PBS for 20 min 4 °C, followed by a washing step with PBS FBS2% EDTA 2 mM azide 0.1% and fixed in PFA 2%. Flow cytometry was performed using the MACSQUANT (Miltenyi Biotec) and data analysis was performed with the FlowJo software.

### Cytotoxicity assay

The cytolytic activity of engineered T-cells endowed with the different CARs (transfected with dose D_1/4_), was assessed using a flow cytometry-based cytotoxicity assay. In this assay target cells presenting the CAR target antigen (Daudi, CD19^pos^) are labelled with CellTraceTM CFSE (Life Technologies) and control cells not presenting the CAR target antigen (K562, CD19^neg^) with CellTraceTM violet (Life Technologies). The target cell populations were co-incubate at 37 °C at various ratio of engineered effector CAR T cells (Effector/Target ratio of 10:1, 20:1 and 30:1) in a final volume of X-Vivo-15 media of 100 uL, for 5 h in presence of vehicle (Ethanol) or AP21967 (100 nM).

The whole cell population was recovered, washed in PBS and labeled with eFluor780 viability marker before being fixed by 2% PFA. Fixed cells were analyzed by flow cytometry to determine their viability.

### Statistical analysis

EC50 values of dose-response assays on rapamycin synthetic analog were computed using the R library drc and a four parameter log-logistic function which has the following formula:


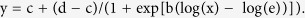


To evaluate the significance of the difference between rapamycin synthetic analog treated and untreated cells for CD19pos cell lysis by CAR T cells expressing the FKBP/FRB*, a standard paired t-test was used on the different transfections.

## Additional Information

**How to cite this article**: Juillerat, A. *et al.* Design of chimeric antigen receptors with integrated controllable transient functions. *Sci. Rep.* **6**, 18950; doi: 10.1038/srep18950 (2016).

## Supplementary Material

Supplementary Information
