# Supplementary material for: Design of chimeric antigen receptors with integrated controllable transient functions
Source: Sci Rep. 2016 Jan 11;6:18950. doi: 10.1038/srep18950 (PMC4707440; doi:10.1038/srep18950)
Supplement: Supplementary Information [file srep18950-s1.pdf]

Supplementary information for:

Design of chimeric antigen receptors with integrated controllable transient functions

Alexandre Juillerat[1], Alan Marechal[2], Jean-Marie Filhol[2], Julien Valton[1], Aymeric Duclert[2], Laurent Poirot[2] and Philippe Duchateau[2]

[1]Cellectis Inc, 430E, 29th street, NYC, NY 10016, USA

[2]Cellectis SA, 8 rue de la croix Jarry, 75013 Paris

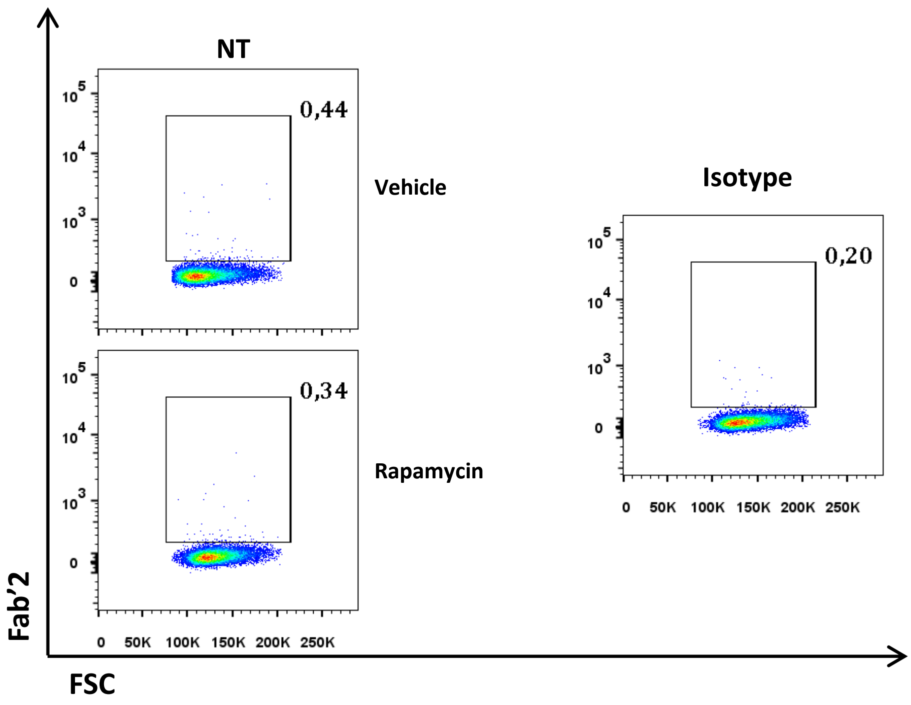

Supplementary figure 1: Percentages of live cells positive for surface detection of mcCAR (non-transfected cells) in function of presence of vehicle (DMSO) or rapamycin. The detection of the Fab'2 region of the scFv is shown in a representative experiment.

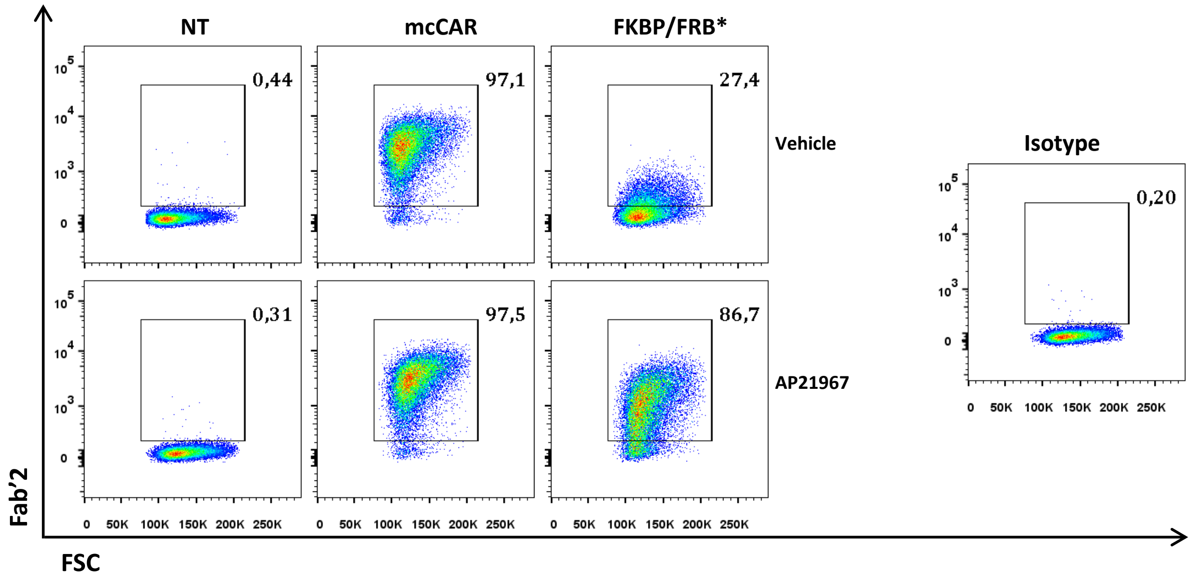

Supplementary figure 2: Percentages of live cells positive for surface detection of mcCAR in function of presence of vehicle (Ethanol) or AP21967. The detection of the Fab'2 region of the scFv is shown in a representative experiment

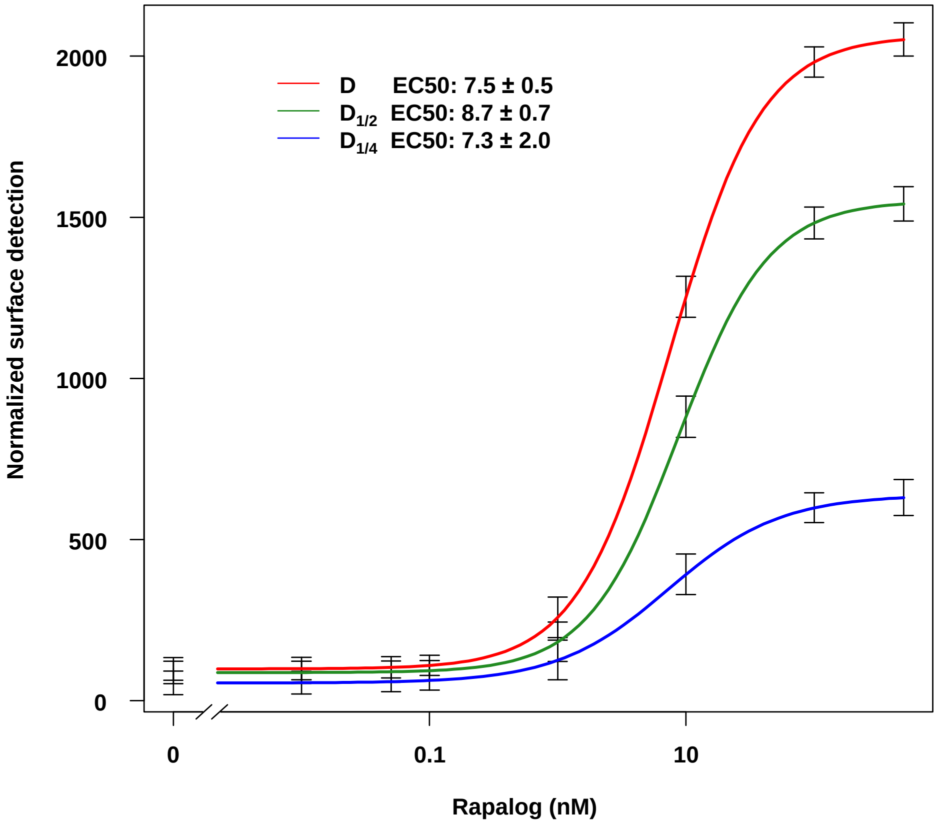

Supplementary figure 3: Characterization of the small molecule switch-on system. Determination of the AP21967 EC50 with CD123 targeting engineered mcCAR. T-cells transfected with three doses (D, $D_{1/2}$ and $D_{1/4}$) of mRNA coding for the engineered mcCAR were treated with increasing amount of AP21967 rapalog. The scFv is detected using a recombinant CD123 fused to an Fc fragment. N=2, error bars denote s.d.

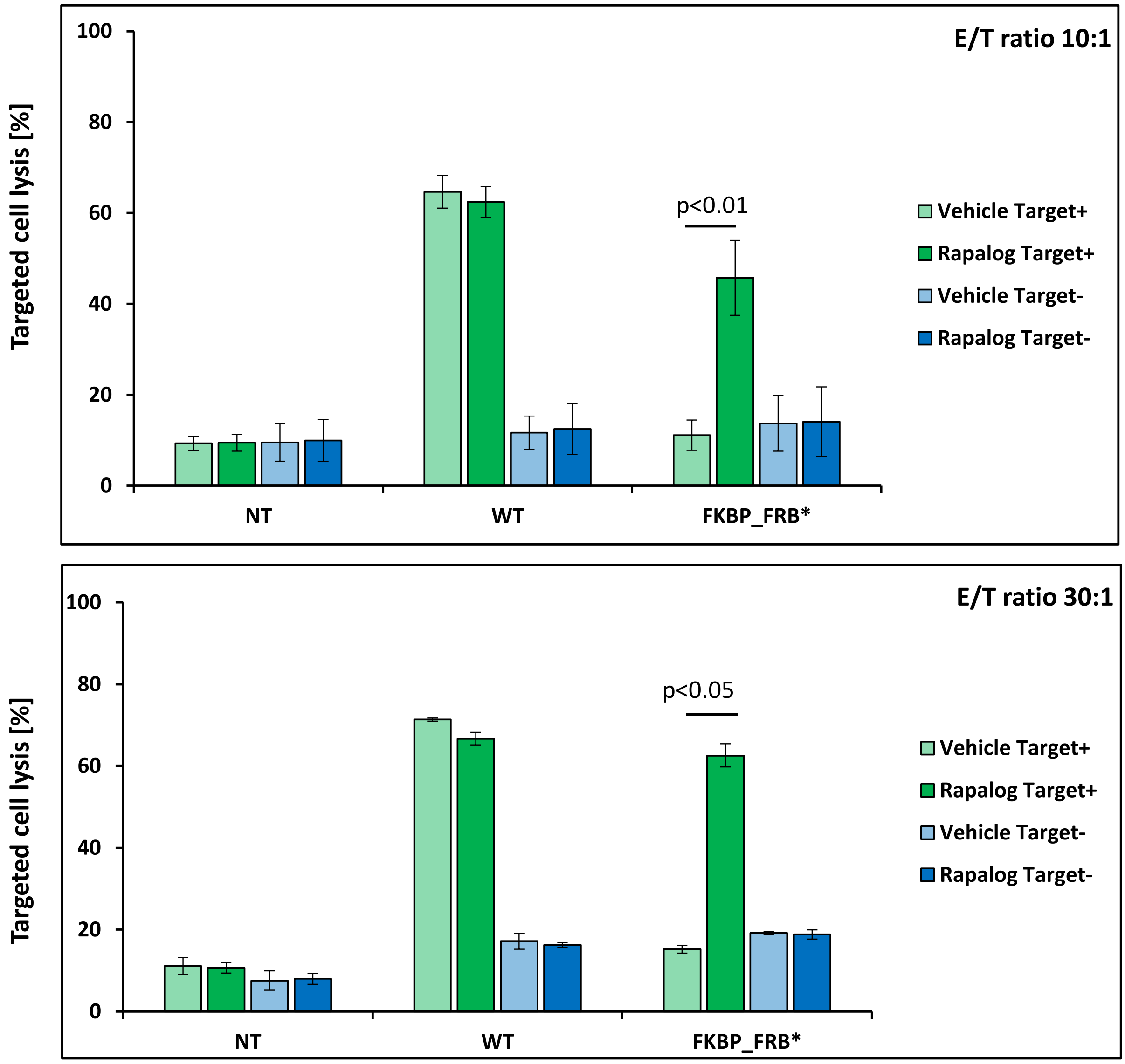

Supplementary figure 4: Cytolytic properties of the engineered T-cells. The effect of the AP21967 rapalog on the cytolyticc capacites of the of the CAR T cells toward model antigen presenting cell was assessed in a flow-based cytotoxicity assay. The $CD19^{pos}$ and a $CD19^{neg}$ target cell viability was measured after coculture with engineered mcCAR T-cells in presence or absence of AP21967. Effector/target ratios was set to 10:1 (upper panel) or to 30:1 (lower panel). NT represents non-transfected T-cells, N=2 for the 30:1 ratio and N=3 for the 10:1 ratio, error bars denote s.d.

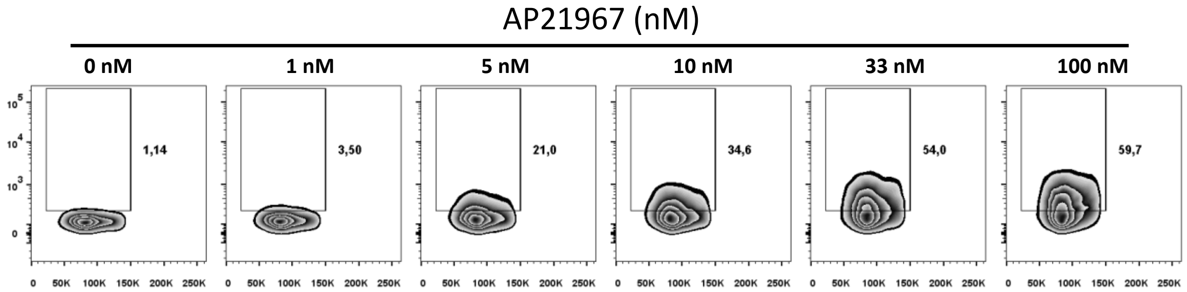

Supplementary figure 5: Percentages of live cells positive for surface detection of FKBP/FRB*-mcCAR in function of presence of increasing dose of AP21967.

**FRB**	RVAILWHEMWHEGLEEASRLYFGERNVKGMFEVLEPLHAMMERGPQTLKETSFNQAYGRDL MEAQEWCRKYMKSGNVKDLTQAWDLYYHVFRRI
**FKBP**	GVQVETISPGDGRTFPKRGQTCVVHYTGMLEDGKKFDSSRDRNKPFKFMLGKQEVIRGWEE GVAQMSVGQRAKLTISPDYAYGATGHPGIIPPHATLVFDVELLKLE
**FRB/FKBP**	RVAILWHEMWHEGLEEASRLYFGERNVKGMFEVLEPLHAMMERGPQTLKETSFNQAYGRDL MEAQEWCRKYMKSGNVKDLTQAWDLYYHVFRRIGSEAAAREAAAREAAAREAAARGVQVET ISPGDGRTFPKRGQTCVVHYTGMLEDGKKFDSSRDRNKPFKFMLGKQEVIRGWEEGVAQMS VGQRAKLTISPDYAYGATGHPGIIPPHATLVFDVELLKLE
**FKBP/FRB**	GVQVETISPGDGRTFPKRGQTCVVHYTGMLEDGKKFDSSRDRNKPFKFMLGKQEVIRGWEE GVAQMSVGQRAKLTISPDYAYGATGHPGIIPPHATLVFDVELLKLEEAAAREAAAREAAAR EAAARGRVAILWHEMWHEGLEEASRLYFGERNVKGMFEVLEPLHAMMERGPQTLKETSFNQ AYGRDLMEAQEWCRKYMKSGNVKDLTQAWDLYYHVFRRI
**FKBP/FRB***	GVQVETISPGDGRTFPKRGQTCVVHYTGMLEDGKKFDSSRDRNKPFKFMLGKQEVIRGWEE GVAQMSVGQRAKLTISPDYAYGATGHPGIIPPHATLVFDVELLKLEEAAAREAAAREAAAR EAAARGRVAILWHEMWHEGLEEASRLYFGERNVKGMFEVLEPLHAMMERGPQTLKETSFNQ AYGRDLMEAQEWCRKYMKSGNVKDLLQAWDLYYHVFRRI

SupplementaryTable 1: Sequences of FRB, FKBP or FRB and FKBP fusions used in this work

**α-chain-F**	GCATCGTAATACGACTCACTATAGGGCAGGCCACCATGGCTCCCGCAATGGAGTC
**α-chain-R**	TCAATTGTTCTTGGGGTTTGGT
**β-chain-F**	GCATCGTAATACGACTCACTATAGGGCAGGCCACCatggacactgagtctaacc
**β-chain-R**	TCACAGCTCACAGCCTCCTT
**γ-chain-F**	GCATCGTAATACGACTCACTATAGGGCAGGCCACCATGATCCCAGCCGTGGTCCT
**γ-chain-R**	TCAGCGAGGGGGCAGGGCCT

SupplementaryTable 2: Sequences of oligonucleotides used to amplify the three different polypeptide chains (alpha, beta and gamma) of the mcCAR for mRNA preparation.
